# A227 EXPLORING THE ROLE OF MICROBIAL AND GENETIC FACTORS IN DEFINING THE INTESTINAL IMMUNE SYSTEM USING A HUMANIZED GNOTOBIOTIC MOUSE MODEL

**DOI:** 10.1093/jcag/gwad061.227

**Published:** 2024-02-14

**Authors:** G Bayer, B K Tsankov, B Samman, E Foerster, N Winsor, G V Visser, S Hakim, E Allen-Vercoe, D Philpott

**Affiliations:** University of Toronto, Toronto, ON, Canada; University of Toronto, Toronto, ON, Canada; University of Toronto, Toronto, ON, Canada; University of Toronto, Toronto, ON, Canada; University of Toronto, Toronto, ON, Canada; University of Toronto, Toronto, ON, Canada; University of Toronto, Toronto, ON, Canada; University of Guelph, Guelph, ON, Canada; University of Toronto, Toronto, ON, Canada

## Abstract

**Background:**

Studies of the gut microbial ecosystem and its role in disease are often complicated by the intricate network of interactions between its microbial members and the host. Therefore, mouse models in which the microbiota diversity can be strictly controlled, and the functional potential be easily assessed, are needed.

**Aims:**

Establishing a taxonomically and functionally diverse human gnotobiotic microbiota that recapitulates many microbe-host interactions of the complex human microbiome. To validate the effects of the defined bacterial gut community on the host, we will assess host physiology, intestinal immunity and the response to inflammatory stimuli.

**Methods:**

Germ-free (GF) mice were inoculated with a defined bacterial community isolated from the stool of a healthy human donor. Stability of colonization across multiple generations and spatial organization of the bacterial community in the intestine were assessed using 16S rRNA gene sequencing and species-specific quantitative PCR. Immune cells were isolated from the colonic lamina propria and characterized by flow cytometry.

**Results:**

Inoculated GF mice were reproducibly colonized with 8 bacterial species, collectively termed Human Defined Community 1 (HDC1). The HDC1 is vertically transmitted from mothers to offspring and the relative abundances remain stable in the colony over an extended period in a gnotobiotic isolator. We observed spatial stratification of the HDC1 microbiota between the small intestine and colon or cecum. HDC1 mice form a colonic inner mucus layer comparable in thickness to SPF mice suggesting physiological spatial organization of the bacterial community over the cross-sectional axis of the colon. Characterization of colonic lamina propria immune cells in HDC1 mice revealed myeloid and lymphoid cell abundances closely resembling those of mice harboring a complex microbiota (specific pathogen-free [SPF] mice), including normal macrophage and dendritic cell counts and normal proportions of CD4^+^ cells (regulatory T, Th1 and Th17 cells). Finally, we showed that HDC1 mice can be transplanted with human gut commensals and Crohn's disease (CD)-associated bacteria, demonstrating the tractability of this human defined microbiota.

**Conclusions:**

In summary, HDC1 gnotobiotic mice are stably colonized with a human defined bacterial community and harbor an intestinal innate and adaptive immune system similar to SPF mice. Therefore, HDC1 mice represent a reliable and tractable tool for host-microbe interaction studies. Ultimately, we will employ the HDC1 model to decipher the role of *Nod2* – the strongest genetic risk factor for CD development – in shaping both the microbiota and host immunity.

**Funding Agencies:**

CIHROGS

